# Jacobsen syndrome and neonatal bleeding: report on two unrelated patients

**DOI:** 10.1186/s13052-021-01108-2

**Published:** 2021-07-01

**Authors:** Gregorio Serra, Luigi Memo, Vincenzo Antona, Giovanni Corsello, Valentina Favero, Paola Lago, Mario Giuffrè

**Affiliations:** 1 Department of Health Promotion, Mother and Child Care, Internal Medicine and Medical Specialties “G. D’Alessandro”, University of Palermo, Palermo, Italy; 2 Clinical Genetics Outpatient Service, Neonatology and Neonatal Intensive Care Unit, San Bortolo Hospital, Vicenza, Italy; 3 Neonatal Intensive Care Unit, Ca’ Foncello Hospital, Treviso, Italy

**Keywords:** JBS, 11q23 deletion, aCGH, Thrombocytopenia, Early diagnosis, Genotype-phenotype correlation

## Abstract

**Introduction:**

In 1973, Petrea Jacobsen described the first patient showing dysmorphic features, developmental delay and congenital heart disease (atrial and ventricular septal defect) associated to a 11q deletion, inherited from the father. Since then, more than 200 patients have been reported, and the chromosomal critical region responsible for this contiguous gene disorder has been identified.

**Patients’ presentation:**

We report on two unrelated newborns observed in Italy affected by Jacobsen syndrome (JBS, also known as 11q23 deletion). Both patients presented prenatal and postnatal bleeding, growth and developmental delay, craniofacial dysmorphisms, multiple congenital anomalies, and pancytopenia of variable degree. Array comparative genomic hybridization (aCGH) identified a terminal deletion at 11q24.1-q25 of 12.5 Mb and 11 Mb, in Patient 1 and 2, respectively. Fluorescent in situ hybridization (FISH) analysis of the parents documented a de novo origin of the deletion for Patient 1; parents of Patient 2 refused further genetic investigations.

**Conclusions:**

Present newborns show the full phenotype of JBS including thrombocytopenia, according to their wide 11q deletion size. Bleeding was particularly severe in one of them, leading to a cerebral hemorrhage. Our report highlights the relevance of early diagnosis, genetic counselling and careful management and follow-up of JBS patients, which may avoid severe clinical consequences and lower the mortality risk. It may provide further insights and a better characterization of JBS, suggesting new elements of the genotype-phenotype correlations.

## Introduction

Jacobsen syndrome (JBS; MIM 147791), also known as 11q23 deletion, is a rare contiguous gene syndrome with about 200 patients reported to date [1]. It has a prevalence estimated around 1 in 100,000 newborns, and a female/male ratio 2:1 [2]. The deletion size ranges from about 7 Mb to 20 Mb, between sub band 11q23.3 and the telomere [3]. The majority of cases (85–92%) has a de novo origin, while 8–15% are caused by a deletion due to parental balanced chromosome translocation, or other rearrangements [3]. Main clinical features are growth (pre- and postnatal) and developmental delay, craniofacial dysmorphisms and blood (thrombocytopenia or pancytopenia) disorders. Malformations of heart, urogenital and gastrointestinal tracts, brain and skeleton may also be observed, as well as ocular, hearing, immunological and hormonal defects [2, 3]. Phenotypic variability depends on type and number of deleted genes [2, 4]. 20% of patients die during the first 2 years of life for complications from congenital heart disease (CHD) or, less commonly, from bleeding [3].

We report on two unrelated newborns with 11q23 deletion syndrome observed in Italy. Our report highlights the importance of early diagnosis, genetic counselling and careful management of JBS patients. Multidisciplinary and long-term follow-up should be guaranteed to these subjects, oriented to avoid or anticipate possible severe clinical consequences (anemia, thrombocytopenia, infections due to immunological deficit), and lower the mortality risk.

## Patients’ presentation

### Patient 1

**Fig. 1:**
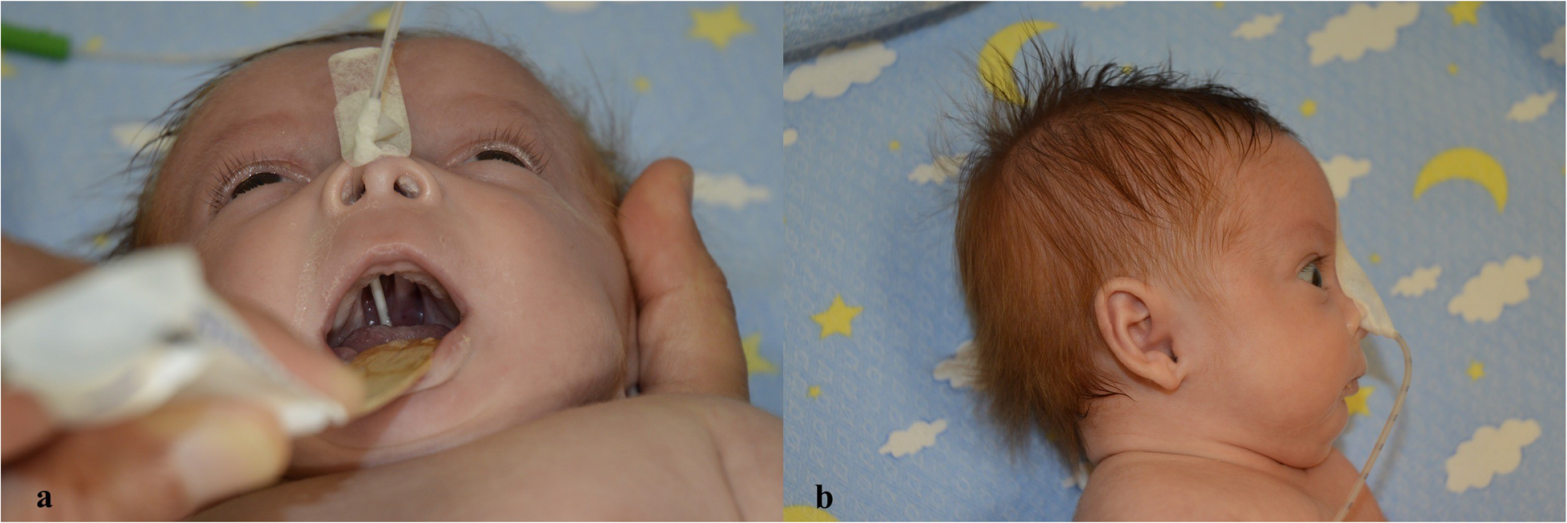
**a/b** Patient 1. **a** cleft palate; **b** lateral view, brachycephaly with flattened occiput, posteriorly rotated ear and retrognathia

**Fig. 2:**
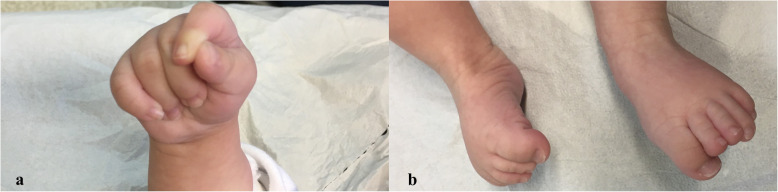
**a/b** Patient 1. **a** clinodactyly of the fifth finger, enlargement and adduction of the thumb; **b** bilateral *pes equinus varus*, and crowded toes

A female newborn, second child of healthy Italian nonconsanguineous parents, was delivered at 37^+ 4^ weeks of gestation by cesarean section for fetal growth restriction (FGR) and polyhydramnios. Apgar scores were 6 and 8, at 1 and 5 min respectively. Anthropometric measurements at birth were as follows: weight 1980 g (1st centile, − 2.37 SD), length 46 cm (12th centile) and occipitofrontal circumference (OFC) 31.3 cm (5th centile, − 1.67 SD). After birth, she suffered from respiratory distress syndrome, which needed noninvasive mechanical ventilation until the 7th day of life. On day 1, cutaneous *petechiae* were observed on the trunk, revealing a subtending thrombocytopenia (platelet count < 50,000/μL) which requested platelet transfusions. During the following 2 days, also anemia and coagulative alterations occurred, requiring red blood cells and plasma transfusions. The subsequent clinical course was characterized by a bacterial infection, treated with intravenous antibiotics until day 13, as well as feeding problems and persistent thrombocytopenia. At 2 weeks of life she was transferred to our Department, to deepen the genetic diagnostic work-up. At admission, physical examination showed: pale skin, multiple hematomas and signs of venipuncture on the limbs, brachycephaly with high prominent forehead and flattened occiput, and facial dysmorphisms (posteriorly rotated ears, ocular hypertelorism, alternating divergent strabismus, broad and flat nasal bridge, anteverted nares, prominent columella, long and hypoplastic philtrum, cleft palate, V-shaped mouth and thin upper lip, retrognathia) (Fig. 1a/b). Finger alterations (bilateral clinodactyly of the fifth one, enlargement and adduction of the thumbs), bilateral *pes equinus varus* and crowded toes were also observed (Fig. 2a/b). Generalized hypotonia and feeding difficulties completed her clinical profile. Abdominal ultrasonography (US) revealed bilateral II degree hydronephrosis (according with radiology grading system), with renal pelvis dilation, in the anteroposterior diameter, of 7 and 10 mm of the left and right kidney respectively. Urine analysis and renal function tests were normal. US heart evaluation showed atrial septal defect (ASD) and patent *ductus arteriosus*.

aCGH analysis (100–150 Kb resolution, genomic assembly GRCh37.p13) identified an 11q24.1-q25 deletion of 12.5 Mb, and indicated the positions 122,293,668 and 134,868,407 as the breakpoints of the rearrangement. The deleted region involved around a hundred genes, including among others *JHY*, *BSX*, *NRGN*, *FEZ1*, *CHEK1*, *ETS1*, *FLI1*, *KCNJ1*, *ARHGAP32*, *ADAMTS8*, *JAM3* and *B3GAT1*. FISH was then performed in both parents, showing normal results and, thus, confirming a de novo origin of the genetic abnormality. The patient recovered from the initial feeding difficulties at around 1 month of life. Hearing screening through transient-evoked otoacoustic emissions (TEOAEs) revealed abnormal results. In order to ascertain and characterize the hearing loss, an audiological assessment was started. It included to date auditory brainstem response (ABR), which showed bilateral mild conductive hypoacusis. During the second month of life thrombocytopenia persisted (platelet count never lower than 30,000/μL, and not associated with other hematological alterations), however without further episodes of bleeding. She was discharged at 2 months of age, and included in a multidisciplinary follow-up. She currently is 5 months old, and her anthropometric measures are: weight 4160 g (<3rd centile), length 58 cm (<3rd centile) and OFC 39 cm (3rd centile) (according to World Health Organization growth chart for neonatal and infant close monitoring) [5]. She has global developmental delay, mild axial hypotonia and hypertonia of the scapular and pelvic girdles. Osteotendinous and archaic reflexes are normal. Blood examination shows thrombocytopenia (86,000/μL) and normal total immunoglobulin levels. Abdominal US reveals a bilateral decrease of calico pelvic dilation to 5 and 6 mm (anteroposterior diameter) of the left and right kidney, respectively.

### Patient 2

A female newborn, second child of healthy nonconsanguineous Eastern european (Ukrainian mother and Estonian father) parents, was delivered at 34^+ 5^ weeks of gestation, by spontaneous vaginal birth for preterm labor. Pregnancy was complicated by threatened miscarriage at 26 and 30 weeks of gestation, leading to steroidal prophylaxis of respiratory distress syndrome. Apgar scores were 9, 9 and 10, at 1, 5 and 10 min respectively. Anthropometric measurements at birth were as follows: weight 1927 g (18th centile), length 41 cm (4th centile) and OFC 32 cm (59th centile). Physical examination showed: turricephaly, high prominent forehead, and facial dysmorphisms (small, low set and posteriorly rotated ears with hypoplastic antihelix, ocular hypertelorism, thinned iris, epicanthal folds, down slanting palpebral fissures, broad nasal bridge, V-shaped mouth). Fifth finger clinodactyly was also observed. Generalized and severe hypotonia completed her clinical profile. Head US documented an area of encephalomalacia, compatible with cerebral parenchymal hemorrhage occurred in the intrauterine life. Heart and abdominal US were normal. Soon after birth, the clinical course was marked by relevant feeding problems, which needed nasogastric tube, and thrombocytopenia, treated with platelet transfusions. A respiratory distress syndrome appeared as well, and required noninvasive mechanical ventilation for the first month, and then exclusive oxygen support for further few days. The following clinical evolution was characterized by persistent thrombocytopenia, lymphocytopenia and hypogammaglobulinemia, however not associated with further episodes of bleeding and/or infections.

aCGH analysis (100–150 Kb resolution, genomic assembly GRCh37.p13) identified an 11q24.1-q25 deletion of 11 Mb, with the positions 123,414,350 and 134,868,420 as the breakpoints of the rearrangement, associated with a 10p15.3p13 duplication of 15 Mb (from 136,145 to 15,415,339, including many genes). The deleted region is smaller than that observed in Patient 1, sparing *UBASH3B*, *CRTAM*, *JHY*, *BSX*, *HSPA8* and *CLMP* genes. Both parents refused further genetic and hematological investigations. The patient did not recover from her feeding difficulties, and needed continuous enteral nutrition by nasogastric tube. Hearing screening and endocrine tests revealed normal results. Conversely, immunological evaluation identified global lymphocytopenia (marked for T cells, and mild for B and NK cells), as well as mild hypogammaglobulinemia (IgA and IgG levels lower than normal range). She was discharged at 2 months of age, and included in a multidisciplinary follow-up. Currently, she is 3 months old and shows normal growth and severe global developmental delay, with deep reduction of alertness and reactivity, poor suction and spontaneous motor activity, impaired swallowing, severe axial hypotonia and increased muscular tone of the limbs.

## Discussion and conclusions

To date, around 25 liveborn patients with 11q23 deletion syndrome diagnosed perinatally have been reported [6], and the present two newborns add to the clinical database. These patients manifest craniofacial dysmorphisms (including skull deformities like macrocrania, high prominent forehead, facial asymmetry, and commonly observed abnormal head shape as trigonocephaly or brachycephaly) [3, 7], growth/developmental delay and brain defects, blood (thrombocytopenia, morphological abnormalities of the platelets, coagulopathy, anemia, lymphocytopenia) and heart disorders [6], widely overlapping those of our newborns. Both present patients had pancytopenia of variable degree (including thrombocytopenia) and neonatal bleeding. In our cases, the presence of characteristic facial dysmorphic features, skull deformities (brachycephaly and turricephaly) and thrombocytopenia/pancytopenia made the diagnostic suspicion, which was confirmed by aCGH analysis.

**Fig. 3:**
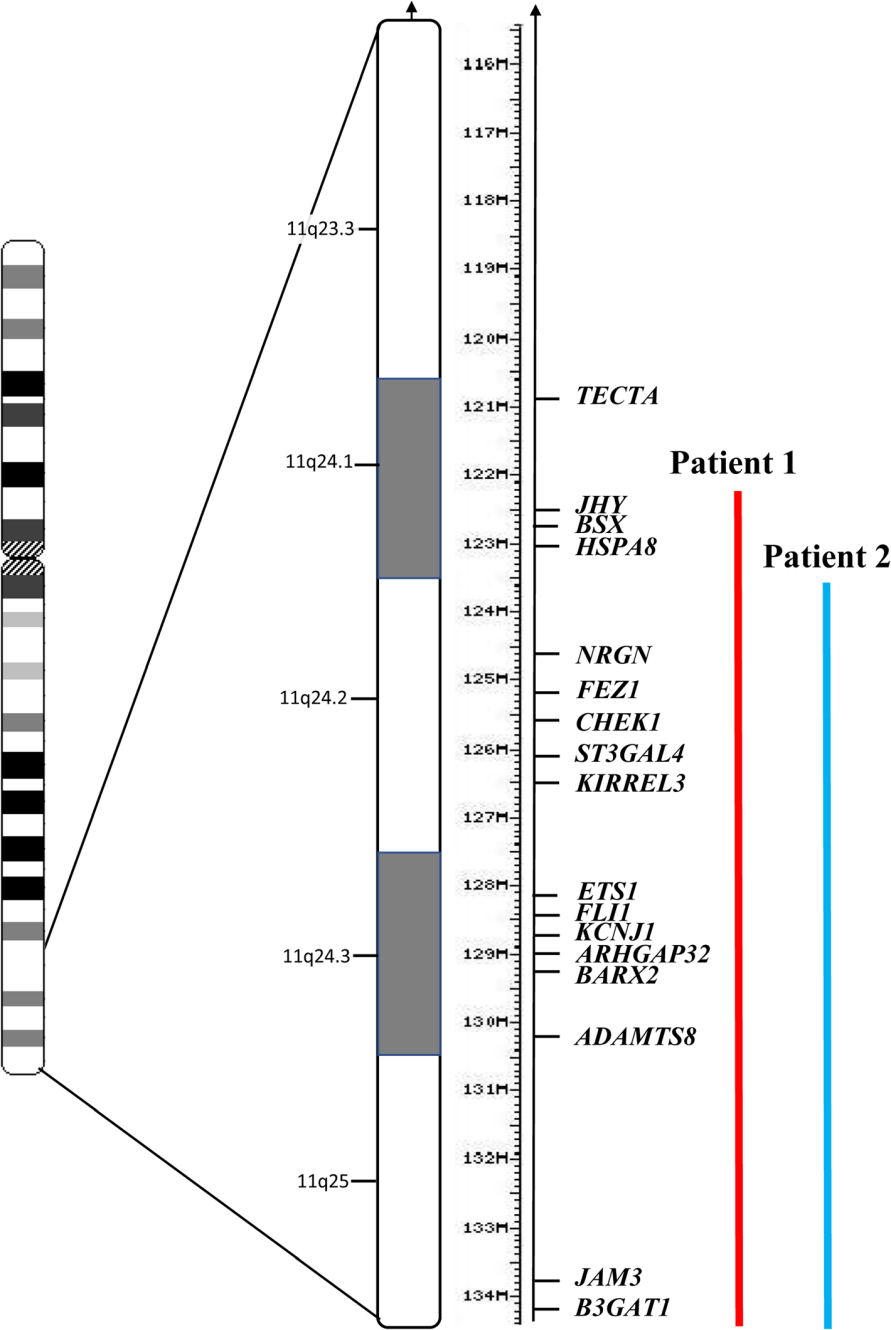
Schema of JBS chromosomal critical region at 11q23.3-qter, and comparison with the deletions of present newborns (red line indicates Patient 1 deletion, blue line Patient 2 deletion)

Although patients with the largest deletions usually show more severe clinical manifestations and neuromotor impairment, the phenotype may widely vary among patients [3]. Indeed, both our newborns presented with high clinical expressivity, and carried deletions (12.5 and 11 Mb) encompassing the majority of JBS critical region (about 14 Mb) and including most of the candidate genes. Patient 2 carried a smaller deletion than that observed in the first newborn, sparing six genes (*UBASH3B, CRTAM, JHY, BSX, HSPA8* and *CLMP*) (Fig. 3). Some of the clinical differences between our two infants may be explained by the different number and type of genes involved [1, 4, 8], as well as by the concomitant presence of different risk factors (FGR, low birth weight, perinatal asphyxia) [6], which may have contributed to variable clinical severity (Table 1). Specifically, among the genetic factors responsible for the clinical variability, the contribution of the subtelomeric trisomy 10p to the phenotype of Patient 2 is not clear [9]. Indeed, there is no evidence of a clearly recognizable 10p duplication syndrome [10]. There are few subjects with “pure” 10p duplication, as most of them carry associated chromosomal anomalies (consequence of unbalanced segregations of familial translocations resulting in trisomy 10p associated with other additional segmental imbalances) [11]. Such patients with 10p13-p15 duplications, without other extra or lost genes, share common features: urinary and genital anomalies, neurological defects (microcephaly, brain anomalies, seizures, motor delay and hypotonia), immunological abnormalities, vision and hearing impairments, skeletal alterations (humped back, abnormal extremities), and cleft lip/palate [10]. However, they have variably shown to date such clinical manifestations [9], and also our Patient 2, indeed, manifests only few of these features (hypotonia, motor delay, hypogammaglobulinaemia). Furthermore, in the present duplicated genomic fragment (spanning 15 Mb at 10p15.3-p13, from 136,145 to 15,415,339) is included *GATA3* gene, whose defects (specifically its deletion) are associated to visual and auditory deficiencies [12], however not currently observed in the proband.

**Table 1:** Comparison of the clinical and genetic features in both patients

	Patient 1	Patient 2
**Physical growth delay**		
Prenatal	yes	Yes
Postnatal	yes	No
**Developmental delay**	yes	Yes
**Feeding difficulties**	yes	Yes
**Craniofacial abnormalities**		
Skull deformities	brachycephaly	turricephaly
High prominent forehead	yes	Yes
**Ears**		
Small ears	no	Yes
Low set ears	no	Yes
Posteriorly rotated ears	yes	Yes
Hypoplastic antihelix	no	Yes
**Eyes**		
Ocular hypertelorism	yes	Yes
Down slanting palpebral fissures	no	Yes
Divergent strabismus	yes	No
Palpebral ptosis	no	No
Epicanthal folds	no	Yes
Iris coloboma	no	no (thinned iris)
**Nose**		
Broad nasal bridge	yes	Yes
Flat nasal bridge	yes	No
Anteverted nares	yes	No
Prominent columella	yes	No
**Mouth**		
Long philtrum	yes	No
Hypoplastic philtrum	yes	No
V-shaped mouth	yes	Yes
Thin upper lip	yes	No
Retrognathia	yes	No
Cleft palate	yes	No
**Hands**		
Fifth finger clinodactily	yes	Yes
Enlarged and adducted thumbs	yes	No
**Feet**		
Crowded toes	yes	No
*Pes equinus varus*	yes	No
**Malformations of the heart**	atrial septal defect	no
**Kidneys**	hydronephrosis	No
**GI tract**	no	No
**Genitalia**	no	No
**Skeleton**	no	No
**CNS**	no	No
**Cerebral US abnormalities**	no	Encephalomalacia
**Hearing loss**	bilateral mild conductive hypoacusis	No
**Immunological defects**	no	lymphocytopenia and hypogammaglobulinemia
**Hematological disorders**	thrombocytopenia and anemia	Pancytopenia
**Hormonal defects**	no	No
**Genetic test result (aCGH)**	11q24.1-q25 deletion(12.5 Mb, position 122,293,668 to 134,868,407)	11q24.1-q25 deletion(11 Mb, position 123,414,350 to 134,868,420)10p15.3p13 duplication (15 Mb, position 136,145 to 15,415,339)

*GI* gastrointestinal, *CNS* central nervous system, *US* ultrasound, *aCGH* array comparative genomic hybridization

Neuromotor impairment, growth delay and facial dysmorphisms may be associated with haploinsufficiency of *ST3GAL4* (ST3 beta-galactoside alpha-2,3-sialyltransferase 4, MIM:104240) [9], *KIRREL3* (kirre like nephrin family adhesion molecule 3, MIM:607761) and *BARX2* (BARX homeobox 2, MIM:604823) genes, while thrombocytopenia and other hematopoietic defects with that of *FLI1* (Fli-1 proto-oncogene, ETS transcription factor, MIM:193067) [2, 3, 13, 14]. They are, indeed, all deleted in both our probands. Specifically, *FLI1* has a key role in megakaryocytes differentiation, and its heterozygous loss is associated with dysmegakaryocytopoiesis and the Paris-Trousseau thrombocytopenia (PTS) in JBS [15]. PTS is characterized by neonatal thrombocytopenia which may resolve over time, and platelet dysfunction (usually persistent), as well as by the presence of two different types of abnormal platelets in peripheral blood (giant platelets and platelets with giant alpha granules) [16, 17]. It has been reported in the majority of JBS patients, affecting at least 88.5% of subjects, and the platelet abnormalities found both in PTS and JBS (and present also in our newborns) have been supposed to be the same condition [3, 18]. This may not be surprising, as JBS is a contiguous gene syndrome. *ETS-1* (ETS proto-oncogene 1, transcription factor, MIM:164720) has an essential role in heart cell migration and development [1, 19]. However, its alteration may be not sufficient to cause cardiac defects, since only Patient 1 (of both newborns bearing its deletion) had CHD. Therefore, heart (ASD) and ear (hypoacusis) involvement in Patient 1 may be associated to genes centromeric than the deleted region of Patient 2. Moreover, *TECTA* (tectorin alpha, MIM:602574) gene, which is spared by the rearrangement in both cases, is suggested to be involved in neurosensorial deafness [3]. Nonetheless, in Patient 1 a mild conductive hypoacusis was found. This may lead to consider other genes (for example among those additionally deleted in Patient 1) in the pathogenesis of hearing loss in JBS patients, and in the meantime to rule out *TECTA* as gene associated to conductive deafness in JBS. The deletion of two genes, *JHY* (junctional cadherin complex regulator, MIM:617594) and *HSPA8* (heat shock protein family A member 8, MIM:600816), highly expressed in the kidneys and deleted in Patient 1 (while they are spared in Patient 2), may explain her renal involvement (hydronephrosis). Conversely, the more severe immunohematological profile of Patient 2, presumably associated to more relevant bleeding and major susceptibility to infections, may be related to the genes included in 10p duplication, as well as to other genetic (regulatory genes, alterations in intronic regions or in the expression of not deleted genes) and epigenetic factors (FGR, prematurity) [20–22]. It is also hard to establish whether her severe neuromotor impairment may be due to the encephalic bleeding likely occurred during the intrauterine life, or if it is the result of an additional deleterious effect on the phenotype of 10p13-p15 duplication, or also if it is a combination of them both.

Our report highlights how in some cases an effective secondary prevention may be challenging and hard to perform (i.e. prenatal bleeding) [2, 6]. Then, early and continuous management (obstetric, perinatal and neonatal), careful follow-up (pediatric cardiologist and neurologist, abdominal US to rule out pyloric stenosis and kidney and urinary tract malformations, ophthalmological, audiological and orthopedic evaluations, blood, immunological and endocrine tests, oncologic surveillance) [3, 23–26], and integrated (occupational, speech, physical and behavioral therapists) home-based care, must be even more necessary for these patients [27, 28], whose complex clinical picture may be worsened by additional concomitant factors [29]. Indeed, when JBS cases are promptly detected (an increasing number may be perinatally suspected and diagnosed, as in present patients), it is recommended a gentle and less invasive perinatal management, including elective cesarean section and avoidance or minimal use of vascular punctures [6]. Although the platelet count may reach low normal values (as documented also in our newborns), a severe bleeding may occur, both in the intrauterine life and postnatally [3, 6]. Clinicians must anticipate and better manage medical and surgery complications (e.g. prophylactic transfusion with platelets and/or whole blood), to reduce the mortality risk [2, 3].

To date, studies performed in JBS led to the identification of genetic modifiers as likely contributors to the phenotypic variability [30], in addition to differences in deletion size. Whole genome sequencing will likely provide further insights into the genetic basis of this variability, and have important implications for therapies [28, 31–33]. Additional risk factors, like those reported in present newborns, must be carefully taken into account and managed by the neonatologist. He must be aware of the potential role of such epigenetic factors, which may increase the risk of complications and worsen clinical evolution and outcome of patients.

## Data Availability

The datasets used and analyzed during the current study are available from the corresponding author on reasonable request.

## Abbreviations

aCGH: Array comparative genomic hybridization
ASD: Atrial septal defect
CHD: Congenital heart disease
FISH: Fluorescence in situ hybridization
FGR: Fetal growth restriction
JBS: Jacobsen syndrome
PTS: Paris-Trousseau syndrome
US: Ultrasonography
